# Soybean miR159*-GmMYB33* Regulatory Network Involved in Gibberellin-Modulated Resistance to *Heterodera glycines*

**DOI:** 10.3390/ijms222313172

**Published:** 2021-12-06

**Authors:** Piao Lei, Nawei Qi, Yuan Zhou, Yuanyuan Wang, Xiaofeng Zhu, Yuanhu Xuan, Xiaoyu Liu, Haiyan Fan, Lijie Chen, Yuxi Duan

**Affiliations:** 1 Nematology Institute of Northern China, Shenyang Agricultural University, Shenyang 110866, China; piaolei9411@163.com (P.L.); 2019200127@stu.syau.edu.cn (N.Q.); zy648794@163.com (Y.Z.); wyuanyuan1225@163.com (Y.W.); syxf2000@163.com (X.Z.); xuanyuanhu007@hotmail.com (Y.X.); liuxiaoyu7805@163.com (X.L.); fanhaiyan6860@163.com (H.F.); chenlijie0210@163.com (L.C.); 2 College of Plant Protection, Shenyang Agricultural University, Shenyang 110866, China; 3 College of Biological Science and Technology, Shenyang Agricultural University, Shenyang 110866, China; 4 College of Sciences, Shenyang Agricultural University, Shenyang 110866, China

**Keywords:** soybean, *Heterodera glycines*, miR159, *GmMYB33*, gibberellin

## Abstract

Soybean cyst nematode (SCN, *Heterodera glycines*) is an obligate sedentary biotroph that poses major threats to soybean production globally. Recently, multiple miRNAome studies revealed that miRNAs participate in complicated soybean-SCN interactions by regulating their target genes. However, the functional roles of miRNA and target genes regulatory network are still poorly understood. In present study, we firstly investigated the expression patterns of miR159 and targeted *GmMYB33* genes. The results showed *miR159-3p* downregulation during SCN infection; conversely, *GmMYB33* genes upregulated. Furthermore, miR159 overexpressing and silencing soybean hairy roots exhibited strong resistance and susceptibility to *H. glycines*, respectively. In particular, miR159-*GAMYB* genes are reported to be involve in GA signaling and metabolism. Therefore, we then investigated the effects of GA application on the expression of *miR159-GAMYB* module and the development of *H. glycines*. We found that GA directly controls the miR159-*GAMYB* module, and exogenous GA application enhanced endogenous biologically active GA_1_ and GA_3_, the abundance of miR159, lowered the expression of *GmMYB33* genes and delayed the development of *H. glycines*. Moreover, SCN infection also results in endogenous GA content decreased in soybean roots. In summary, the soybean miR159-*GmMYB33* module was directly involved in the GA-modulated soybean resistance to *H. glycines*.

## 1. Introduction

Plant-parasitic nematodes (PPNs) are major threats to plant health and pose great concern to food security globally. The most economically significant species have incredible abilities to modify the physiology and immunity of their hosts. PPNs are called the hidden enemies of agricultural production, which cause enormous losses estimated over 100 billion USD per year [1,2,3]. Among them, soybean cyst nematode (SCN, *Heterodera glycines*) is a sedentary obligate biotroph and causes more than $1.2 billion and $120 million in annual yield losses in the United States and China, respectively, making it the most harmful pathogen of soybean [4,5]. Briefly, the second-stage juveniles (J2) of soybean cyst nematode hatch from the eggs directly penetrate soybean roots using their needle-like stylet to puncture the cell wall [6]. Once the J2s arrive in the vascular cylinder, they select a single root cell and deliver secretions into the root cell to reprogram the expression of plant genes to their advantage. Finally, a complex permanent feeding site called syncytium is induced [7,8]. When the syncytium established, the J2s become sedentary and start absorbing nutrients from the syncytium to favor their development. Undergoing several moults, J2s develop into to mature male and female [9]. Cyst nematode has evolved multifaceted strategies to suppress their hosts’ defence system and maintain their feeding sites to effectively complete their life cycle. Much of this complex parasitism process requires the involvements of small RNAs, especially microRNAs [10,11,12].

Plant small RNAs (sRNAs) are a class of short regulatory RNAs that have a length of 20–24 nucleotides. According to differences of precursors, biogenesis, and modes of action of different sRNAs, they are classified into three major types. The microRNAs (miRNAs) are derived from single-stranded hairpins as hairpin RNAs (hpRNAs), and phased siRNAs (phasiRNAs) are generated from miRNA target transcripts. Small interfering RNAs (siRNAs) are derived from double-stranded RNA (dsRNA) precursors [13,14]. The biogenesis of miRNAs starts from the transcription of *MIR* genes, which are mainly located in intergenic regions. Firstly, the *MIR* gene is transcribed into primary miRNA(pri-miRNA) by RNA polymerase II (Pol II). Then, pri-miRNA is processed into precursor miRNA (pre-miRNA) by DCL1. Furthermore, HUA ENHANCER 1 (HEN1) converts pre-miRNA into short miRNA/miRNA* duplex. Next, one strand of the miRNA/miRNA* is selected and loaded by ARGONAUTE 1 (AGO1) to form an RNA-induced silencing complex (RISC) [15,16]. RISC is guided by the almost perfect sequence complementary of miRNA and target mRNA to induce post-transcriptional gene silencing (PTGS) by cleavage (slicing) or translational repression. Finally, miRNAs regulate diverse biological processes in growth regulation and biotic and environmental stress responses [17,18].

The first evidence of plant’s miRNAs responses to PPN infection comes from the Arabidopsis-*H. schachtii* module [12]. For instance, miR160 and its target genes *AtARF10/16/17* (*Auxin responsive factor 10/16/17*) exhibited opposite expression patterns during the infection of *H. schachtii*. Other miRNAs and corresponding target genes, such as miR167-*AtAP2* (*APETALA 2*), miR396-*AtGRF1/3/8* (*growth regulating factor 10/16/17*), miR398-*AtCSD1/2*(*Cu/Zn superoxide dismutase 1/2*) and miR858-*AtMYB83* were also found differently expressed in Arabidopsis-*H. schachtii* interaction [19,20]. Owing to the advances of accuracy in deep sequencing, the expression patterns of various soybean miRNAs were identified in response to soybean cyst nematode infection, including miR159, miR172, miR398 and miR408 [10,11]. However, these research studies only offered limited microRNAs profiles. The roles of most miRNAs and their target genes in soyben-SCN interaction remain to be experimentally confirmed.

To date, only a few research studies elucidated the function of miRNAs and their target genes in plant–PPNs interaction. For example, the expression of the miR396-*GRF* regulatory module resulted in decreased syncytium size and arrested nematode development in Arabidopsis. This observation was also further validated in soybean, and homeostasis of the soybean miRNA396-*GRF* network is essential for *H. glycines* development and reproduction [19,21]. The miR319-*TCP4* (*TEOSINTE BRANCHED1/CYCLOIDEA/PRO-LIFERATING CELL FACTOR 4*) module was demonstrated to have a function in the interactions of tomato and root-knot nematode. Overexpression of miR319 decreased the amount of JA in tomato and reduced the resistance against root-knot nematode *Meloidogyne incognita* [22]. Arabidopsis *mir390* mutant lines infected with *M. javanica* showed a significant decrease in the infection rate and size of the RKN induced gall [23]. Moreover, miR172 was found upregulated in the gall formed by *M. javanica* in Arabidopsis. Further investigation found that the miR172/TOE/FT regulatory module played a negative role in Arabidopsis resistance to *M. javanica* [24]. Recently, the roles of another important conserved miRNA159 and its target *GAMYB* genes of Arabidopsis were also studied during the parasitism of *M. incognita* by Medina et al. Their results showed that the Arabidopsis *mir159abc* mutant line had lower susceptibility to RKN, and *MYB33*::GUS (β-glucuronidase) expression was observed within galls at early stages of gall development [25]. miR159 was also demonstrated to play essential roles in other plant species against pathogens. For example, cotton and Arabidopsis accumulate miR159 in response to the fungus, *Verticillium dahlia* [26]. Since the miR159 family is highly abundant, strongly conserved and ubiquitously targeted *GAMYB* genes in all eudicots and monocotyledonous plants have been sequenced [27]. It is not surprising that the miR159-*GAMYB* module has been reported to play significant roles in programmed cell death (PCD) in the aleurone and tapetum of Arabidopsis seed [28], flower time delay [29], strawberry fruit development [30] and grapevine floral development [31]. *GAMYB* genes are a kind of R2R3 MYB transcription factor that function in the gibberellin (GA) pathway. Therefore, much of miR159-*GAMYB* module-regulated biological processes were accompanied by gibberellin signal transduction [32].

Several previous studies have found soybean miR159 family members differently expressed during SCN parasitism [10,11,33,34]. The evidence from high-throughput sequencing implicated that the soybean miR159-*GAMYB* module may play an important role in soybean resistance to SCN. In this study, firstly, we conducted in silico analyses of soybean miR159 family m4w3q4w3qembers and their target *GAMYB* genes. Then, the expression patterns of miR159 family members and their target *GAMYB* gene response to soybean cyst nematode infection were investigated. miR159 overexpressing and silencing transgenic soybean hairy roots showed strong resistance and susceptibility to *H. glycines*, respectively, due to miR159 and *GAMYB* genes that have been reported to be regulated by GA in other plant species [28,31]. Therefore, we investigated the effect of GA application on expression of miR159 and found a positive correlation between GA and miR159. Our present study may contribute to a better understanding of miR159 in soybean–SCN interaction as well as the relationship between GA and miR159.

## 2. Results

### 2.1. Soybean miR159 Family Members Are Responsive to Heterodera glycines Infection

Previous research studies reported that soybean miR159 family members respond to *Heterodera glycines* infection in early and late parasitic stages [10,11,33]. In order to figure out how soybean miR159 family members respond to SCN. We analyzed the number and distribution of miR159 in the soybean genome. Six soybean pre-miR159 sequences were found in an miRbase, namely pre-miR159a-f (Supplementary Table S1). The sequence pre-miR159d was part of pre-miR159a in the same genomic location; thus, pre-miR159d was removed from our present research. Totally, there were five canonical *MIR159* genes. Phylogenetic analysis showed that all precursors of fives soybean *MIR159* genes belong to two subfamilies: subfamily one, pre-miR159a and e; subfamily two, pre-miR159b, c and e (Supplementary Figure S1A). The stem-loop structures of pre-miR159 family members were folded by using RNAfold wrapper in TBtools. All but pre-miR159c formed miR159-5p/miR159-3p (miR159-5p/miR159-3p*) duplex structures (Supplementary Figure S1B), and the mature sequences of each miR159 subfamily were almost identical, differentiated by the 3’-most nucleotide(s) (Supplementary Table S1).

**Figure 1:**
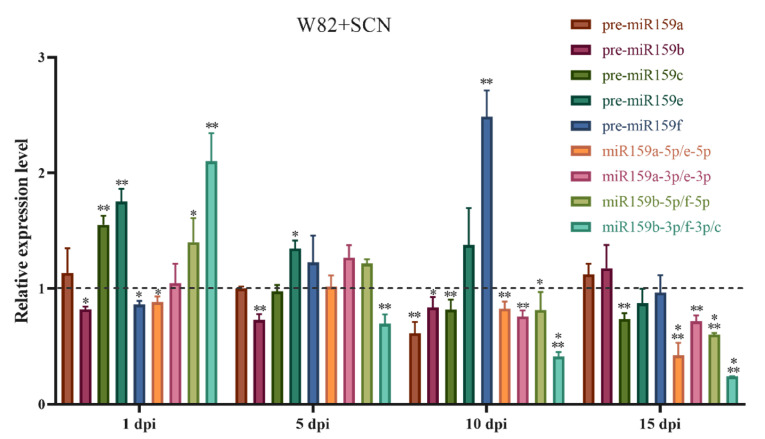
Time-course expression analysis of soybean miR159 during soybean cyst nematode (SCN) infection with susceptible cultivar Williams 82. dpi, post-inoculation. The expression of precursors and miR159 members in Williams 82 inoculated with SCN (W82 + SCN) and was relative to Williams 82 control (W82CK) at each time point. Soybean *U6* gene was used as internal reference gene. Statistical comparisons were made with the *t*-test for every soybean miR159 member and precursors between W82 and W82 + SCN at each time point. Significant differences between each comparison found at *p* < 0.05, *p* < 0.01 and *p* < 0.001 level were marked with *, ** and ***, respectively.

Based on mature miR159 sequences information, we designed primers to detect temporal expression patterns of soybean miR159 family members by qRT-PCR in the susceptible soybean cv. Williams 82 (W82) roots inoculated with soybean cyst nematode. All the precursors and mature sequences of miR159 were expressed in the W82 roots when infected by *H. glycines* (Figure 1). At 1 dpi (day post inoculation), the expressions of pre-miR159a, pre-miR159c, pre-miR159e and miR159b-3p/f-3p/c were upregulated, while pre-miR159b, pre-miR159f and miR159a-5p/e-5p were repressed. At 5 dpi, pre-miR159b and miR159b-3p/f-3p/c significantly downregulated (*p* < 0.01), and there were no significant changes in the expression levels of other genes at *p* < 0.05 level. All genes but pre-miR159f were severely decreased in expression level at 10 dpi. At 15 dpi, all mature sequences of miR159 and pre-miR159c were significantly downregulated (Figure 1).

### 2.2. GAMYB Genes Were Up-Regulated in Response to H. glycines Infection

**Figure 2:**
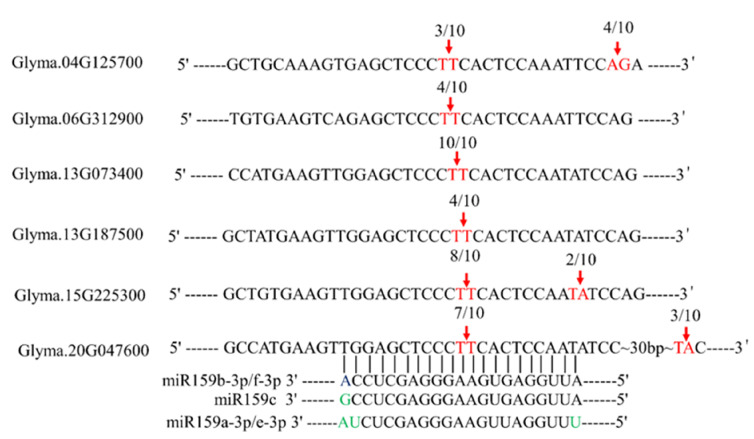
Validation of the cleavage sites of miR159 target genes. The soybean mature miR159 sequences were placed into psRNATarget (http://plantgrn.noble.org/psRNAtarget/, accessed on 1 February 2020) to predict their target genes and cleavage sites with default parameters, followed with a 5′-RACE PCR experiments that were conducted to confirm the cleavage sites of miR159. Ten different clones for each target gene 5′-RACE PCR products were analyzed by DNA sequencing. The cleavage positions were highlighted with red arrows, the cleavage frequency is shown above and the characters in green colour indicate different nucleotides of mature miR159 sequences.

In plants, microRNAs suppress their target genes to control diverse biological processes. miR159 is reported to target R2R3 MYB transcription factors, referred to as *GAMYB*s or *GAMYB*-likes (*GAMYB*Ls) [32]. The abundant mature sequences were miR159-3p of miR159 family members described in miRbase and other plant species [32]. We set miR159-3p sequences as our primary research objectives. To verify whether soybean miR159 targets *GAMYB* genes, we firstly predict all the potential target genes in psRNATarget (http://plantgrn.noble.org/psRNAtarget/, accessed on 1 February 2020) [35] with default parameters. Then, soybean *GAMYB* genes were screened from the predicted results, six putative *GAMYB* genes were predicted as the target genes of miR159 family members: Glyma.04G125700, Glyma.06G312900, Glyma13G73400, Glyma.13G187500, Glyma15G225300 and Glyma.20G47600 (Figure 2). Putative miR159 family members target sites were identified in the transcripts of all above-predicted genes. Based on these results, 5’-RACE experiments were conducted to confirm the cleavage sites predicted. Albeit variable, the DNA sequencing results of 5’-RACE products showed that all soybean miR159 members can cleavage putative soybean *GAMYB* genes predicted (Figure 2).

**Figure 3:**
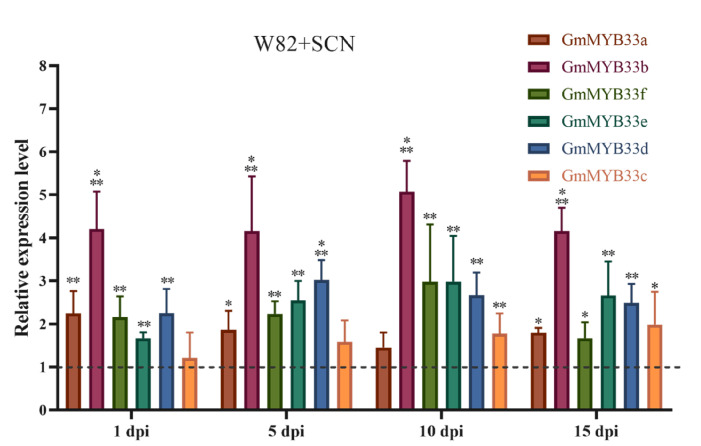
Soybean *GmMYB33* genes were upregulated during the soybean parasite by *H. glycines*. dpi, day post inoculation. Expression levels of *GmMYB33* in W82-innoculated SCN were relative to W82 control. *Gmubi-3* was used as internal reference gene. Error bars represent the standard deviation from the means. *t*-test was applied to compare the differences of expression level between W82 control and W82 inoculated with SCN at each time point. SCN, soybean cyst nematode. *, ** and ***, significant difference found at *p* < 0.05, *p* < 0.01 and *p* < 0.001 levels.

In Arabidopsis, miR159 family members are reported to suppress the expression of *MYB33*, *MYB65* and *MYB101* to regulate plant growth, development and programmed cell death [36,37]. To identify putative soybean *GAMYB* genes, we conducted a phylogenetic analysis of putative target *GAMYB* genes. According to their position in the phylogenetic tree. We renamed Glyma.04G125700, Glyma.06G312900, Glyma13G73400, Glyma.13G187500, Glyma15G225300 and Glyma.20G47600 as *GmMYB33*c, *GmMYB33*d, *GmMYB33*b, *GmMYB33*e, *GmMYB33*f and *GmMYB33*a, respectively (Supplementary Figure S2). Subsequently, we examined the expression level of soybean *GmMYB33* genes in response to the infection of *H. glycines*. Interestingly, almost all *GmMYB33* genes were upregulated during the entire SCN infection period. Strikingly, the expression level of *GmMYB33*b was the highest among *GmMYB33* genes and upregulated four times compared with W82 control (*p* < 0.001). The expression of *GmMYB33*a, *GmMYB33*c, *GmMYB33*e, *GmMYB33*d and *GmMYB33*f also activated and shared similar expression patterns under SCN-infected conditions in soybean roots (Figure 3). These results suggested that the miR159-*GmMYB33* regulatory network is involved in *H. glycines* infection.

### 2.3. miR159 Positively Regulates Soybean Resistance to H. glycines

**Figure 4:**
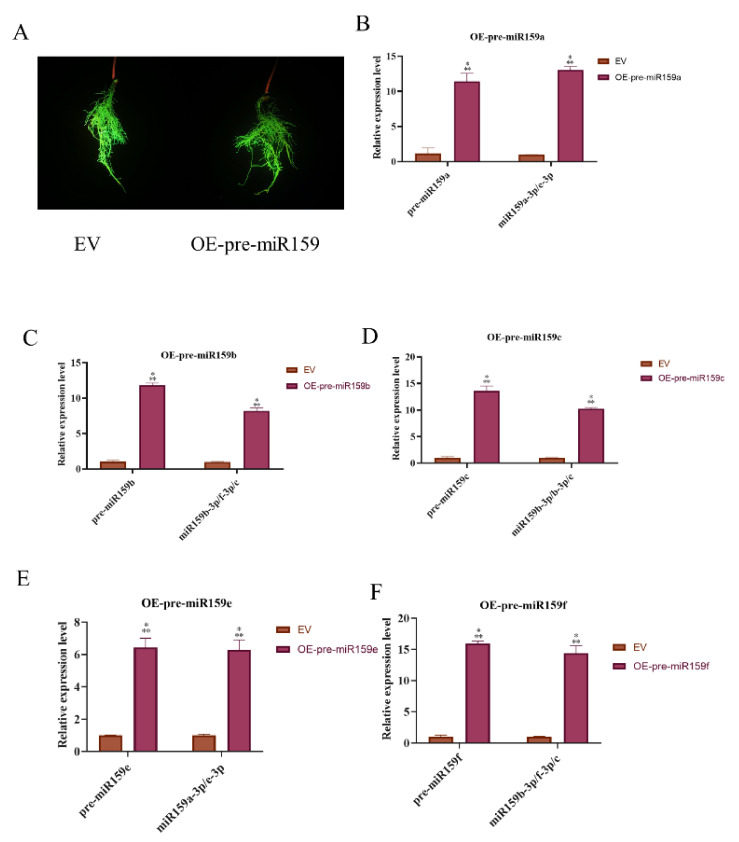
The expression level of miR159 in transgenic pre-miR159 overexpressing soybean hairy roots. (**A**) Soybean hairy roots with strong GFP signal, EV, hairy roots of empty vector, OE-pre-miR159 and miR159 overexpressing soybean hairy roots. (**B**–**F**) The relative expression level of precursors and mature sequences of miR159 detected by qRT–PCR. Expression levels of miR159 in OE were relative to EV with soybean *U6* as the internal reference gene. *t*-test was applied to compare differences between OE and EV hairy roots. ***, significant difference found at *p* < 0.001 level. Error bars represent the standard deviation from the means.

**Figure 5:**
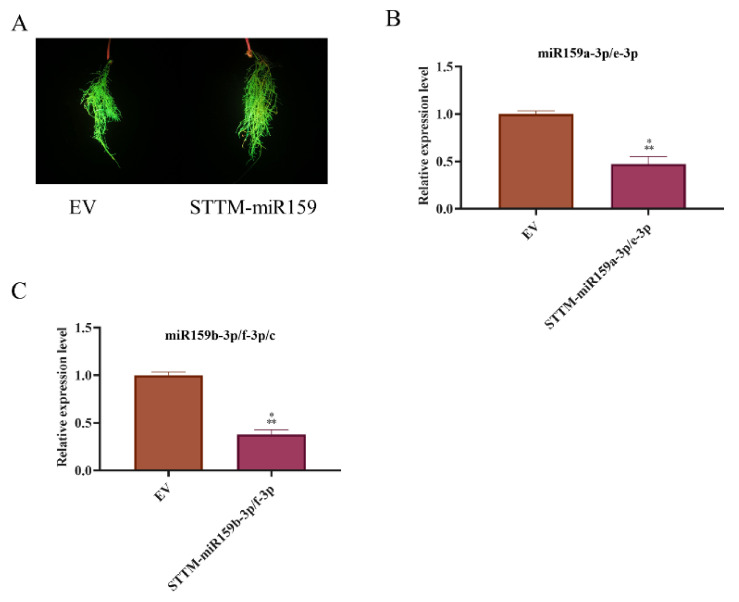
The expression level of miR159 in transgenic STTM-miR159 soybean hairy roots. (**A**) Soybean hairy roots with strong GFP signal, EV, hairy roots of empty vector, STTM-miR159, soybean hairy roots of short tandem target mimic (STTM) of miR159. (**B**,**C**) The relative expression level of mature sequences of miR159 detected by qRT–PCR. Expression levels of miR159 in STTM were relative to EV with soybean *U6* gene as the internal reference gene. *t*-test was applied to compare differences between STTM and EV hairy roots. ***, significant difference found at *p* < 0.001 level. Error bars represent standard deviation from the means.

In order to determine the roles of miR159 family members in soybean-SCN interaction. We constructed overexpressing and silencing (STTM-159) plasmids control by the *Gmubi* promotor (Figures S3 and S4) to generate soybean hairy roots. Soybean hairy roots with strong GFP signals were used to assay the development of SCN and expression levels of miR159 family members (Supplemetary Supplementary Figure S5). Under the transcriptional control of the *Gmubi* promotor, the relative expression level of precursors and mature sequences of miR159 in the hairy roots harboring overexpressing plasmids strikingly increased compared with hairy roots harboring an empty vector (EV) from six to fifteen times (Figure 4). Conversely, the relative expression level of mature sequences of miR159 in soybean hairy roots expressing STTM-miR159 decreased dramatically compared with EV (Figure 5).

**Figure 6:**
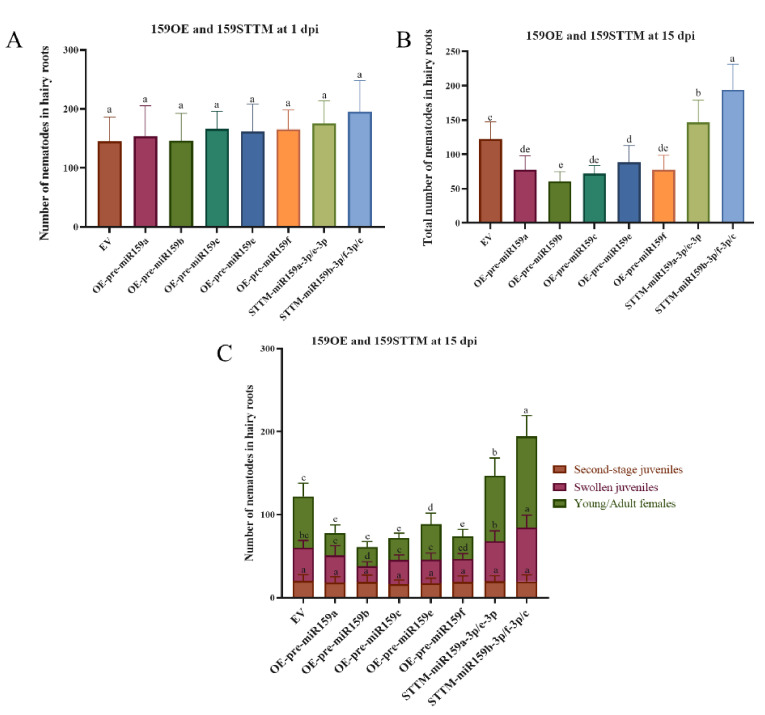
Penetration and development of soybean cyst nematode in transgenic hairy roots. (**A**) Number of second-stage juveniles in hairy roots examined at 1 dpi. (**B**) Total number of nematodes in hairy roots at 15 dpi. (**C**) Number of different stages nematodes in the soybean hairy roots. EV, empty vector; OE-pre-miR159, miR159 overexpressing soybean hairy roots; STTM-miR159, soybean hairy roots of short tandem target mimic (STTM) of miR159. dpi, day post inoculation. Multiple statistical comparisons between nematodes in EV and other transgenic hairy roots were made by one-way analysis of variance (ANOVA) followed by LSD post hoc test. Different characters mean significant differences found at *p* < 0.05 level. ns, not significant. Error bars represent standard deviation from the means.

This result indicated that our overexpressing and silencing plasmids could efficiently enhance and reduce the abundance of miR159 in transgenic soybean hairy roots. Furthermore, these transgenic soybean hairy roots were used to determine the role of miR159 in soybean–SCN interaction by investigating penetration and development at 1 and 15 dpi, respectively. In order to evaluate the development of SCN juveniles in a very consistent manner, we applied a three-grade scoring system consisting of second-stage juveniles (vermiform-J2, Supplementary Figure S6A), swollen juveniles (swollen J2/J3/J4, Supplementary Figure S6B–D), young females and mature females (Supplementary Figure S6E,F). At 1 dpi, there were no differences in the number of penetrating *H. glycines* second-stage juveniles found in EV, miR159 overexpressing and silencing soybean hairy roots (Figure 6A). At 15 dpi, the total number of nematodes significantly decreased in all pre-miR159 overexpressing soybean hairy roots compared with the empty vector, while it was increased in miR159 silencing (Figure 6B). Moreover, overexpressing all precursors of miR159 delayed the development of SCN juveniles; the portion of swollen juveniles and young/adult females decreased significantly compared with the empty vector, and an opposite phenomenon was observed in the miR159 silencing hairy roots (Figure 6). Therefore, soybean miR159 family members positively regulate soybean resistance to *H. glycines* by inhibiting the development of the nematode, rather than reducing the number of J2s that enter the vascular cylinder to the parasite.

**Figure 7:**
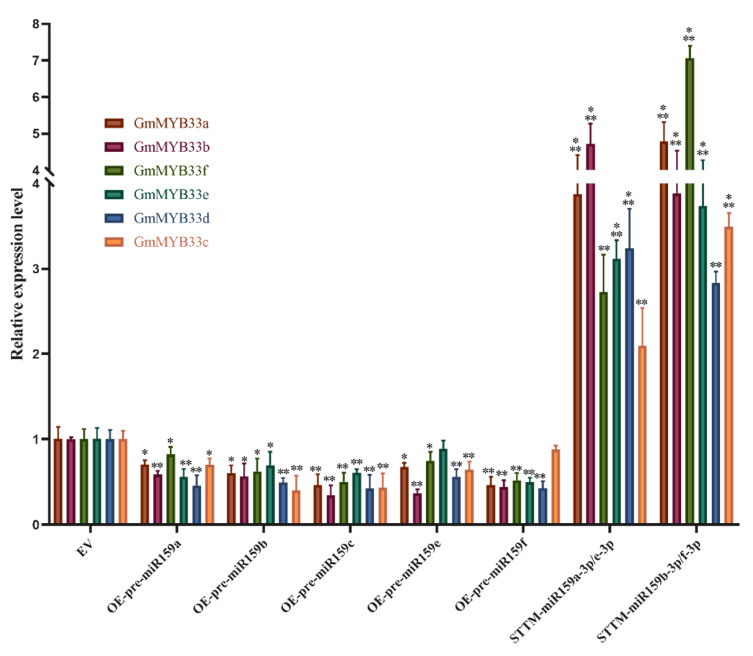
The expression level of *GmMYB33* genes in miR159 overexpressing and silencing soybean hairy roots. Error bars represent standard deviation from the means. The expression levels of *GmMYB33* in OE and STTM were relative to EV with *Gmubi-3* as internal reference gene. *t*-test was applied to compare the expression level difference between EV control and miR159 overexpressing, EV and silencing soybean hairy roots; OE-pre-miR159, miR159 overexpressing soybean hairy roots; STTM-miR159, soybean hairy roots of short tandem target mimic (STTM) of miR159. *, ** and ***, significant difference found at *p* < 0.05, *p* < 0.01 and *p* < 0.001 levels.

Furthermore, we examined the expression level of miR159 targeting genes *GmMYB33* in both miR159 overexpressing and silencing soybean hairy roots. As expected, the expression level of all *GmMYB33* genes downregulated in miR159 was found to be overexpressed. Conversely, *GmMYB33* genes upregulated two to seven times in silencing soybean hairy roots (Figure 7).

### 2.4. Gibberellin Activate miRNA159 in Soybean Roots

**Figure 8:**
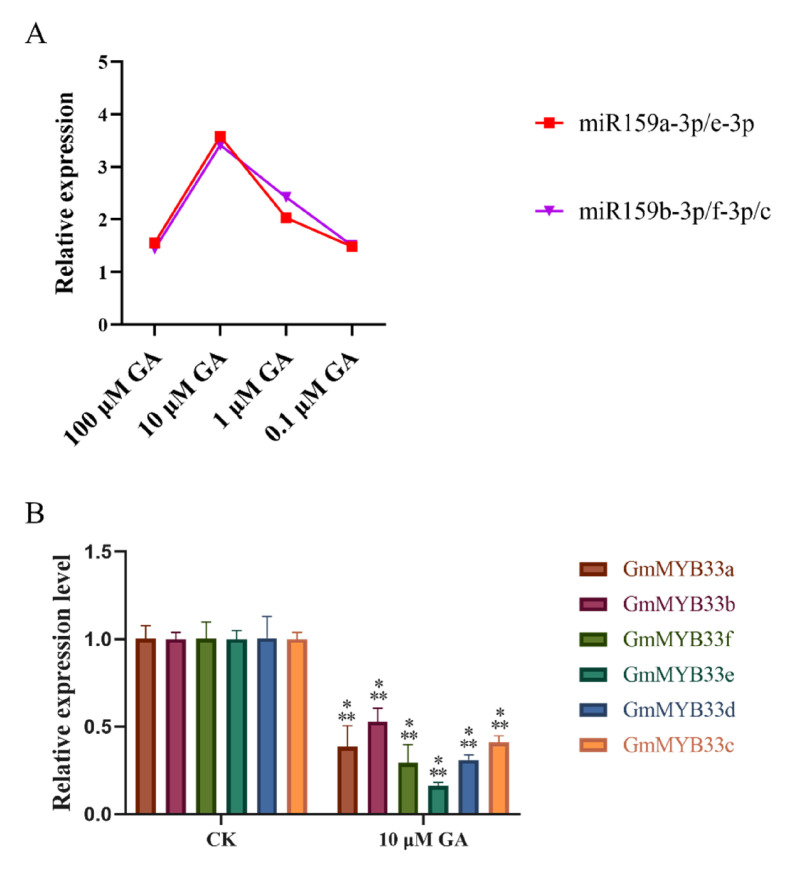
The responses of soybean miR159 family members and *GmMYB33* genes to different concentrations of GA treatments. (**A**) The expression trend of soybean miR159a-3p/e-3p and miR159b-3p/f-3p/c. (**B**) The expression patterns of *GmMYB33* genes under 10 μM GA_3_ condition. Expression levels of miR159 and *GmMYB33* genes in GA-treatments were relative to control treatment with soybean *U6* gene and *Gmubi-3* as internal reference genes, respectively. ***, significant difference found at *p* < 0.001 level. Error bars represent standard deviation from means.

The expression of miRNA159 is controlled by gibberellin (GA) in numerous plant species [32]. To our knowledge, how GA affects the expression of soybean miR159-*GmMYB33* regulatory network is largely unknown. Therefore, we applied four different concentrations of GA_3_ to treat ten-day-old soybean seedlings roots, and short-term (12 h after GA_3_ treatment) effects of GA_3_ application on the expression of soybean miR159 family members and *GmMYB33* genes were evaluated by qRT-PCR. Firstly, we examined the responses of soybean miR159 family members. At all different concentrations GA_3_ induced the abundance of miR159, especially in 10 μM GA_3_ treatment, nearly four times compared with the control treatment, followed by two to three times in 1 μM GA_3_ and 1.5 times in 100 μM GA_3_ and 0.1 μM GA_3_ (Figure 8A). According to this result, we evaluated next the expression patterns of *GmMYB33* genes under 10 μM GA_3_ condition. As expected, exogenous GA application resulted in a significant downregulation in the expression levels of all the *GmMYB33* genes (Figure 8B). The expression of *GmMYB33*e dropped the most, about 0.2 times compared with control. These results suggest that GA could enhance the abundance of soybean miR159 and reduce the abundance of *GmMYB33* genes.

**Figure 9:**
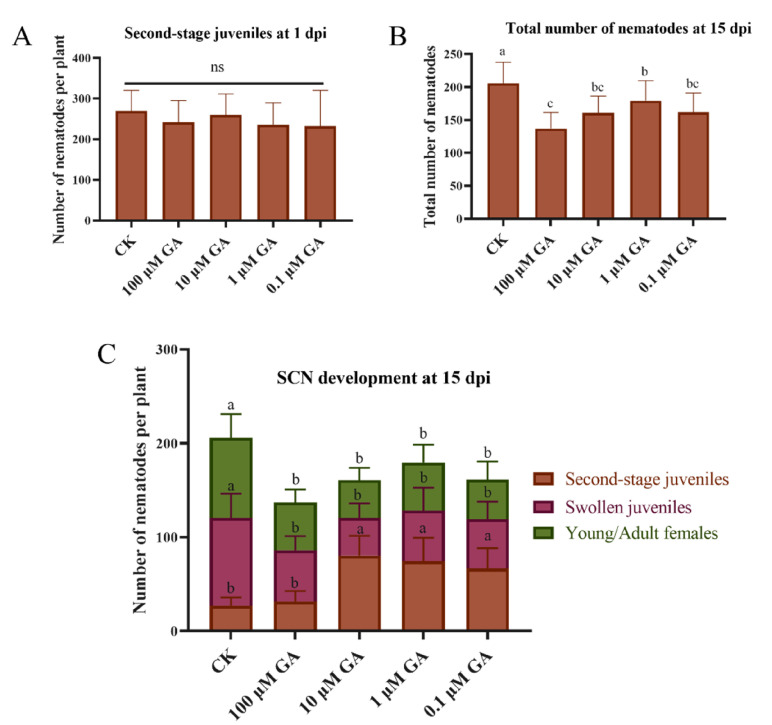
The effects of different concentrations GA_3_ on the development of SCN. (**A**) Penetration assay at 1 dpi. (**B**) Total number of nematodes counted at 15 dpi. (**C**) Different stage nematodes counted at 15 dpi. Multiple statistical comparisons between nematodes in control roots and GA treated roots were made by one-way analysis of variance (ANOVA) followed by the LSD post hoc test. Different characters mean significant differences found at *p* < 0.05 level. ns, not significant. Error bars represent the standard deviation from the means.

Given that soybean miR159 positively regulated soybean resistance to SCN, subsequently, we investigated the effects of different concentrations GA_3_ on the development of SCN. At 1 dpi, no differences were found in the penetration rate of second-stage juveniles of *H. glycines* between control and GA treatments (Figure 9A). All GA-treated soybean roots resulted in significant reductions in the total number of *H. glycines* at 15 dpi, from thirteen to thirty-four percent less than control (Figure 9B). Further analysis found that the portion of second-stage juveniles in control soybean roots was significantly less than 10 μM GA_3_, 10 μM GA_3_ and 0.1 μM GA_3_ treated roots. In addition, SCN juveniles developed into swollen and young/adult females in all GA_3_ treated soybean roots were significantly reduced compared with control roots.

**Figure 10:**
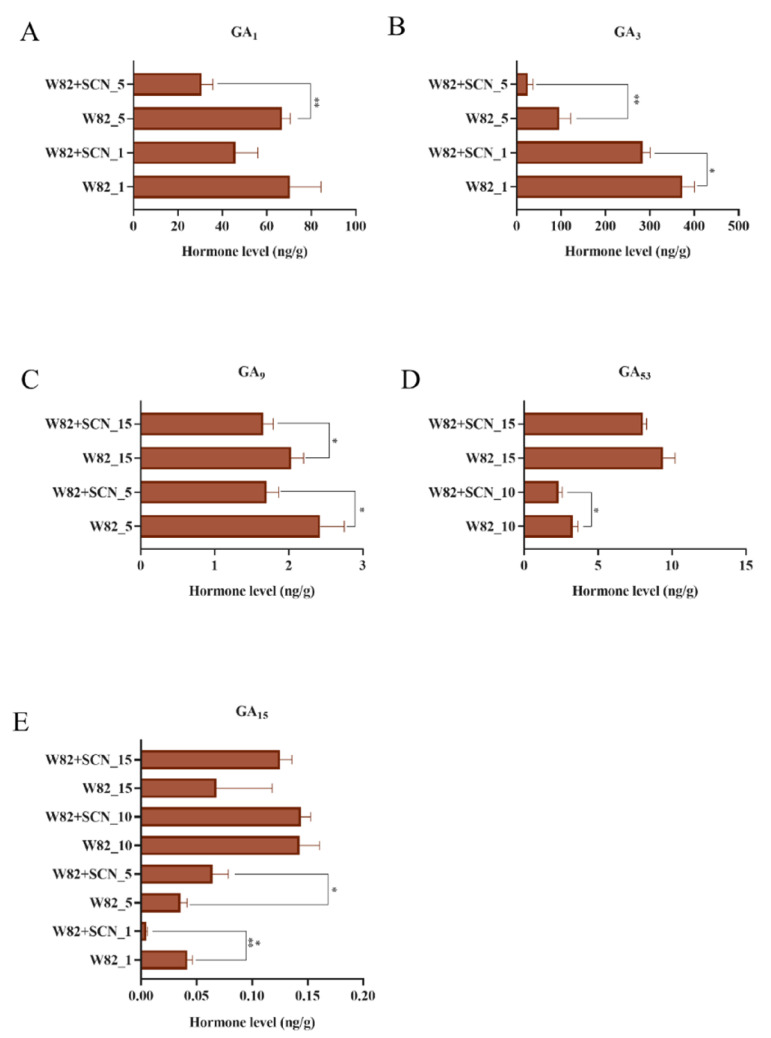
Endogenous GAs content changes in soybean cultivar Williams 82 roots infected by the SCN. (**A**–**E**) Content of GA_1_, GA_3_, GA_9_, GA_53_ and GA_15_ in soybean roots. W82_1, W82_5, W82_10 and W82_15. Williams 82 control at 1, 5, 10 and 15 dpi, likewise, W82 + SCN_1, W82 + SCN_5, W82 + SCN_10 and W82 + SCN_15. Williams 82 inoculated with SCN at 1, 5, 10 and 15 dpi. dpi, day post inoculation. *t*-test was applied to detect the differences of GA content. Error bars represent the standard deviation from the means. *, ** and ***, significant difference found at *p* < 0.05, *p* < 0.01 and *p* < 0.001 level.

Since GA_3_ positively regulated soybean resistance to SCN, we next examined the endogenous GAs content in soybean roots infected by the SCN at 1, 5, 10 and 15 dpi. In total, five GAs were found significantly changed in our study, GA_1_, GA_3_, GA_9_, GA_53_, and GA_15_. Among them, GA_1_ and GA_3_ are biologically active [37]. Their content was quite different; for example, GA_1_ and GA_3_ varied from 30 to 70 ng/g, 25 to 373 ng/g in soybean root, respectively. GA_9_ varied from 1.65 to 2.42 ng/g. GA_53_ varied from 2.32 to 9.38 ng/g. GA_15_ content varied from 0.004 to 0.142 ng/g. GA_1_ and GA_3_ content significantly decreased at 1 dpi and 5 dpi (Figure 10A,B). GA_9_ decreased at 5 dpi and 15 dpi, and GA_53_ decreased at 10 dpi and 15 dpi (Figure 10C,D), while GA_15_ content decreased at 1 dpi and increased at 5 dpi. No significant changes were found at 10 and 15 dpi (Figure 10E).

**Figure 11:**
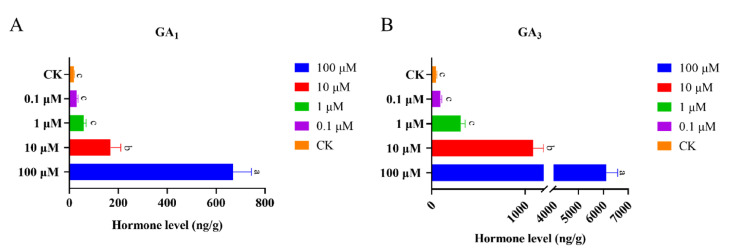
Endogenous GA_1_ and GA_3_ content changes in different concentration GA_3_ treated soybean roots. (**A**,**B**) Content changes of GA_1_ and GA_3_ in the soybean cultivar Williams 82 roots treated with 0.1, 1, 10 and 100 μM GA_3_. Multiple statistical comparisons between nematodes in control roots and GA treated roots were made by one-way analysis of variance (ANOVA) followed by LSD post hoc test. Different characters mean significant differences found at *p* < 0.05 level. Error bars represent standard deviation from the means.

Due to the exogenous application of GA_3_ suppression in the development of SCN, the expression of soybean miR159 was activated, and we wonder how the endogenous biologically active form of GA_1_ and GA_3_ content changes in response to exogenous applications of GA_3_. As shown in Figure 11, both endogenous GA_1_ and GA_3_ content changed dramatically in responding to different concentrations of GA_3_ compared with control, and endogenous GA_1_ varied from 18, 29, 57, 168 and 699 ng/g in CK, 0.1, 1, 10 and 100 μM GA_3_, respectively. Similarly, endogenous GA_3_ varied from 44, 92, 311, 1086 and 6111 ng/g in CK, 0.1, 1, 10 and 100 μM GA_3_, respectively (Figure 11). These results indicated exogenous GA_3_ application upregulated the abundance of endogenous and biologically active forms of GA_1_ and GA_3_; then, endogenous GA_1_ and GA_3_ continued to enhance the expression levels of soybean miR159.

### 2.5. Exogenous GA Application Recovers Soybean Resistance to SCN in the miR159 Silencing Soybean Hairy Roots

**Figure 12:**
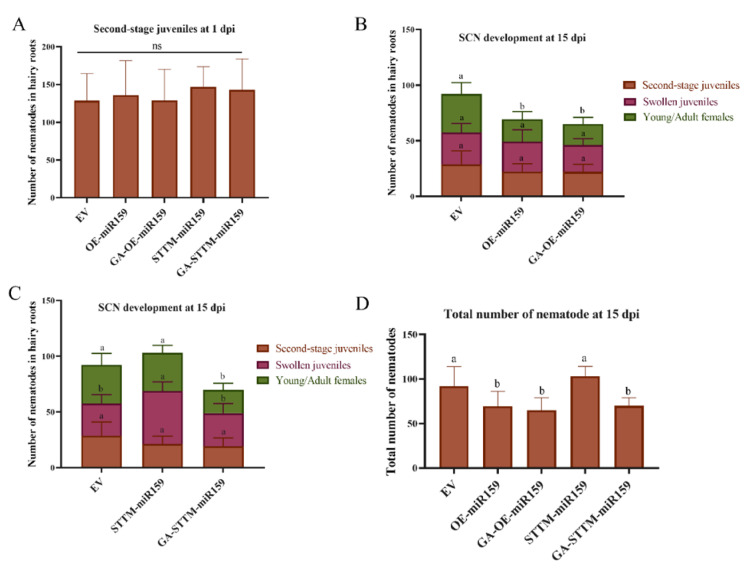
Exogenous GA application recovered soybean resistance to SCN in miR159 silencing soybean hairy roots. (**A**) Penetration assay at 1 dpi. (**B**) Total number of nematodes counted in empty vector, miR159 overexpressing and 10 μM GA_3_ treated miR159 overexpressing hairy roots at 15 dpi. (**C**) Different stage nematodes counted in empty vector, miR159 silencing and10 μM GA_3_ treated miR159 silencing hairy roots at 15 dpi. (**D**)Total number of nematodes in all soybean hairy roots at 15 dpi. Multiple statistical comparisons between nematodes in control roots and GA treated roots were made by one-way analysis of variance (ANOVA) followed by LSD post hoc test. Different characters mean significant differences found at *p* < 0.05 level. ns, not significant. Error bars represent standard deviation from the means.

By considering these results above, we deduced that GA positively regulated soybean resistance to SCN, which may occur through the miR159-*GmMYB33* regulatory network. In order to verify this pathway, we conducted an exogenous 10 μM GA_3_ application on soybean miR159b overexpressing and silencing hairy roots to investigate the development of soybean cyst nematode. GA application had no effects on the penetration of SCN juveniles at 1 dpi (Figure 12A). GA application did not alter the resistance to SCN in the miR159 overexpressing hairy roots, and no significant changes were found in both the total number of nematodes and portions of swollen juveniles and young/adult females (Figure 12B,D). However, in the miR159 silencing soybean hairy roots treated with GA, the total number of nematodes significantly decreased compared with EV and STTM-miR159 (Figure 12C,D). The portion of swollen juveniles and young/adult females reduced significantly between STTM-miR159 and GA treated STTM-miR159 (Figure 12C,D). It indicated that GA application could recover the resistance to SCN in miR159 silencing soybean hairy roots, and GA induced resistance to SCN through the miR159-*GmMYB33* regulatory network.

## 3. Discussion

Plant-parasitic nematodes have striking abilities in manipulating the hosts’ immunity and metabolism to promote their parasitism [38,39]. Since the first plant miRNAome responding to nematode infection was reported in Arabidopsis with *H. schachtii* (beet cyst nematode, BCN) [12]. Multiple miRNAome of host plants analyses have been conducted and used to decipher these molecular mechanisms behind PPNs interaction with their hosts [10,11,22,25]. However, the specific roles of the plant miRNAs and gene regulatory networks are still poorly understood in these complicated interactions. To date, only a few recent studies contributed to our understanding of the functions of conserved plant miRNAs acting in nematode stress responses. For instance, Hewezi et al. found that miR396 downregulation following *GRF1/GRF3* induction was necessary for correct syncytia initiation of *H. schachtii* [19], and a similar functional role of soybean miR396-*GRF* modules was observed in soybean [21]. Cabrera et al. demonstrated that regulatory module miR390-*TAS3* is necessary for proper gall formation and possibly through auxin-responsive factors [23]. Our previous study also found several conserved plant miRNAs differently expressed at the early stage of SCN infection in soybean roots, including miR159. Other studies also found that miR159 was significantly downregulated in different soybean cultivars at different SCN infection stages [10,11,33,34]. These results suggested that miR159 family members may respond to SCN differently. Our next investigation of the temporal expression of miR159 family members in susceptible cultivar Williams82 inoculated with SCN also confirmed that miR159 family members expressed differently at different stages (Figure 1). This phenomenon also was documented in Arabidopsis-RKN interactions. For example, miR159b was downregulated in galls formed by *M. javanica* at 3 dpi [23]. In contrast, Medina et al. and colleagues found miR159a and c were upregulated at 7 dpi and 14 dpi in the gall formed by *M. incognita*. In addition, miR159 was expressed differently in other plants–pathogens interactions; for instance, in *Lilium regal* infected with *Botrytis elliptica*, *Ire-miR159a* expressed differently in resistant and susceptible cultivars, and the expression level of *lre-miR159a* in resistant lines decreased before 24 h post inoculation (hpi). However, *lre-miR159a* slightly changed in susceptible cultivar during the infection period, except for a significant increase at 12 hpi [40,41]. In rice infected with *Magnaporthe oryzae*, the expression of *Osa-miR159a* reduced in a susceptible cultivar at 12, 24 and 48 hpi, while it was increased in a resistant cultivar at 24 hpi [42]. In our study, the expression level of soybean miR159 decreased in response to *H. glycines*, especially at 10 and 15 dpi. Despite miR159 family members differently expressed under cyst nematode and root-knot nematode stress, interestingly, it seems that plant miR159b tends to be downregulated in nematode susceptible plants.

miR159 targets a family of genes encoding R2R3 MYB transcription factors known as “*GAMYB*” or “*GAMYB*-like” transcription factors via a cleavage mechanism [32]. The miR159-*GAMYB* regulatory module was validated by degradome experiments in many species, including eudicots, such as Arabidopsis [43], soybean [44], cotton [45], monocots, wheat [46] and rice [47]. In our present study, miR159-3p sequences were used to predict their targeting genes at psRNATarget. Among these target genes, six putative *GAMYB* genes were screened. Subsequently, the 5’-RACE experiment also confirmed that miR159-3p sequences could cleavage the transcripts of six *GAMYB* genes (Figure 2). These results are also supported by the soybean degradome experiments of Zhou et al. In their findings, all six *GAMYB* transcripts could be cleavaged by miR159-3p sequences [48]. In Arabidopsis, *AtMYB33*, *AtMYB65* and *AtMYB101* were reported to be cleaved by *ath-miR159* [28]. Therefore, according to the positions of the six *GAMYB* genes in the phylogenetic tree (Supplementary Figure S3), a closer evolutionary relationship was observed with *AtMYB33*; therefore, they are renamed *GmMYB33*a/b/c/d/e/f in our study (Supplementary Figure S3). *GAMYB* genes were reported to function as immunity regulators in several plants such as rice [42] and Arabidopsis [49]. However, transcriptome analyses showed that soybean *MYB* genes expressed differently in both SCN resistance and susceptible cultivars [50,51]. In present study, the expression of all *GmMYB33* genes was enhanced under SCN stress. It was consistent with *LrGAMYB* expression patterns in the initial stage of *L. regal*-*B. elliptica* interaction [41].

Several studies have revealed that miR159 plays crucial roles in abiotic stresses, such as drought [52] and heat [53]. In plant–pathogens interactions, *lre-miR159a* plays a positive role in resistance to Botrytis by suppressing the *LrGAMYB* gene [41]. This finding was consistent with our miR159 overexpressing and silencing phenotype. To determine the functional roles of soybean miR159 family members, we constructed miR159 overexpressing and silencing plasmids, and the expression cassettes were controlled by *Gmubi* promotor. According to SCN development data in the present study, overexpressing soybean miR159 enhanced resistance to SCN, and miR159 silencing decreased resistance to SCN. However, in Arabidopsis, an opposite function of miR159 in Arabidopsis-*M. incognita* was observed, and the *mir159abc* mutant had lower susceptibility to *M. incognita* [25]. All these research studies elucidated the essential roles that miR159 played in plant–pathogens interaction.

Gibberellins are a large class of tetracyclic diterpenoids involved in regulating a wide range of biological plants. For instance, GA treatment results in enhanced resistance to the bacterial pathogen *Pseudomonas syringae* pv. *tomato* DC3000 in Arabidopsis but jeopardized resistance against the necrotrophic fungus *Alternaria brassicola* [54]. Different roles of GA were also observed in plant–PPNs interaction. When compared to uninfected control roots, the downregulation of genes involved in GA catabolism was observed in both gall and giant cells induced by *M. graminicola* in rice [55,56]. Exogenous application of GA antagonizes jasmonate-induced rice defense against *M. graminicola* [57]. However, GA enhances rice resistance to migratory root nematode *Hirschmanniella oryzae* [58]. It seems that GA has a varied function in plant immunity depending on the host and pathogen [59].

This contradictory phenomenon was observed in soybean–SCN interaction. Li et al. found that the exogenous application of 100 μM GA_3_ enhanced the resistance of both SCN resistant cultivar Huipizhi and susceptible cultivar Liaodou 15 [51]. On the other hand, a contrary result was reported in a very recent study. Dong et al. revealed that exogenous application reduced the resistance of hairy roots generated from resistant soybean cultivar Peking [60]. According to their description, detached Peking hairy roots were treated with four different concentrations of GA_3_ for 24 h before HG 2.5.7-type SCN inoculation, and the results showed GA increased the ratio of (J3 + J4 + csyts/J2+ J3 + J4 + csyts), and significant differences were observed between control (0 μM GA_3_) and GA treatments (0.1, 1, 10 μM GA_3_) at *p* < 0.01 level. In this study, the exogenous application of GA_3_ enhanced and recovered resistance to *H. glycines* by arresting the development of SCN in wild type W82 roots and STTM-miR159 soybean hairy roots (Figure 9 and Figure 12). Perhaps the different methodsused to generate hairy roots, soybean cultivar and the methods of GA application contributed to opposite results. More experiments need to be conducted to investigate the role of GA in soybean–SCN interaction, such as GA inhibitors, GA related mutant lines, different SCN races (HG type) and different soybean cultivars need to be used to figure out the role GA played in soybean resistance to SCN in future studies.

miRNAs have been identified as important regulators of phytohormone response pathways in plants, influencing their metabolism, distribution and perception [61]. In response to biotic stresses, miRNAs control various phytohormone signaling pathways, as well as several transcription factors (TFs) and defense-related genes [62]. So far, only a few studies revealed that miRNA participated in phytohormone signaling pathways and changed resistance to PPNs. In tomato–RKN interaction, Zhao et al. demonstrated that the miR319-*TCP4* module is engaged in systemic defensive responses, which influenced JA synthesis genes and the endogenous JA levels in leaves, therefore promoting RKN resistance. Their findings showed that miR319 played a role as a systemic signal responder, and regulation of the RKN systemic defense response was mediated by JA [22]. In Arabidopsis–*M. javanica* interaction, the miRNA172/TOE1/FT regulatory module, regulated by auxins, plays a vital role in proper GCs and gall formation [24].

As the GA positively regulates the expression of miR159 in Arabidopsis [29] and grape [31], our findings also confirmed this regulation; 0.1, 1, 10 and 100 μM GA_3_ enhanced the expression of all miR159 mature sequences. Simultaneously, GA deduced the expression level of *GmMYB33* genes dramatically (Figure 8). GA treatment has different effects on miR159 family members. Wang et al. demonstrated that exogenous GA upregulates *VvmiR159c* levels by bypassing DELLA-mediated repression, while no significant changes of the expression of miR159a and b were observed [31]. In strawberry, GA treatment clearly downregulated miR159b and had barely no effects on miR159a [63]. However, in our present results, all soybean miR159 mature sequences were upregulated by GA treatment. Despite expression differences in miR159 family members, given the similar expression of miR159 and target *GAMYB* genes in soybean and grape, we deduced that a similar mechanism of GA regulated by miR159 may exist in both soybean and grape, and soybean miR159-*GmMYB33s* are the essential part of GA signal transduction. Furthermore, we investigate the effects of exogenous application of 10 μM GA_3_ on soybean miR159b overexpressing and silencing hairy roots, and the resistance to SCN recovered and verified that GA controls miR159-*GmMYB33* regulatory networks.

We also found that the endogenous GA content decreased significantly in soybean roots infected with SCN, including two biological active forms, GA_1_ and GA_3_ (Figure 10). This finding was consistent with Li et al., who also found that endogenous GA content decreased significantly in both resistant cultivar Wuzaiheidou and susceptible cultivar Hefeng 35 under the infection of SCN race 3 [64]. In addition, plant growth-promoting rhizobacterium *Bacillus simplex* strain Sneb545 was reported to promote soybean resistance to SCN by activating PR genes and other defense mechanisms [65]. Kang et al. conducted metabolomic analyses and found that the endogenous GA content of soybean was enhanced significantly by Sneb545. Moreover, soybean treated by Sneb545 had the highest content of GA compared with the other three treatments (sterilized water treated soybean, sterilized water treated soybean inoculated SCN and Sneb545 treated soybean inoculated SCN) at 5, 15 and 25 dpi. More importantly, GA content decreased significantly in sterilized water treated soybean inoculated SCN compared with treated soybean [66]. This result indicated GA may also function in the induced systemic resistance (ISR) of soybean. Taken together, these findings indicated that the endogenous GA content of different soybean cultivars may change similarly in responding to SCN infection.

## 4. Materials and Methods

### 4.1. Soybean Germination and Heterodera glycines Juveniles’ Collection

The seeds of soybean (*Glycine max*) cultivar Williams82 (W82) that are susceptible to *H. glycines* were provided by the Nematology Institute of Northern China (NINC, Shenyang, China). These W82 seeds were surface-sterilized with chlorine gas in a fume hood for six hours and then washed several times with distilled water. Finally, W82 seeds were planted in sterilized vermiculite wetted with Hoagland’s nutrient solution and left to germinate in a climatic chamber (light/dark = 16/8, 23–26 °C, 50% relative humidity). Ten days old seedlings of W82 were transferred to PVC tubes containing equal ratios of sterilized sand and soil waiting for SCN inoculation.

The SCN race 3 populations were propagated on susceptible cultivar Williams 82 for two months; then, SCN eggs were extracted from the SCN infested soil and purified using 35% (*w*/*v*) sucrose solution. Eggs were sterilized for 10 min with 0.1% NaClO and then washed several times with sterilized water to eliminate any residues of NaClO. Finally, the sterilized eggs hatched in a modified Baermann pan with 3 mM ZnSO4 solution at 25 °C in the dark for 5 days, and highly active second-stage juveniles (J2s) were collected and used for inoculation [67,68].

### 4.2. Construction of Silencing and Overexpressing Plasmids of Soybean miR159

Firstly, to express our target sequences and visualize the positive transgenic soybean hairy roots, we synthesized the expression part of pG2RNAi2 (GenBank: KT954097, from 452th to 4450th nucleotide, 5′ end flanked with BspEⅠ and 3′ end flanked with PmlⅠ restriction enzyme sites), which contains *Gmubi* promotor, GUS (β-glucuronidase) linker, rcbS terminator and an EGFP (enhanced Green Fluorescent Protein) expression cassette in Genscript (Genscript, Nanjing, China). Then, the synthesis part was inserted into pCMBIA3301 by restriction digest clone. The new binary plasmid was named pNINC2RNAi. Meanwhile, we created a derivative of pNINC2RNAi named pNINC2EX by digesting the GUS-linker out as Noon et al. described [69]. To overexpress all soybean miR159 family members, the precursor sequences of soybean miR159 members were cloned by using Williams 82 gDNA as a template with PrimeSTAR Max DNA polymerase (Takara, Dalian, China); the PCR products were further cloned into T-Vector pMD-19 and verified by DNA sequencing in Sangon (Sangon, Shanghai, China). Then, precursor sequences of soybean miR159 were subcloned into pNINC2EX. To silence miRNAs, we designed short tandem target mimics (STTM) of miR159 family members with the approach that Yan et al. described [70]. STTM159 consists of two short identical sequences that mimic miR159 target sites with three additional nucleotide CTA bulges corresponding to positions 10 to 11 of the miRNA159. Two short identical sequences were linked with 48 nt linker and flanked with AscⅠ and AvRⅡ restriction enzyme sites and at 5′end and 3′ end, respectively, which were synthesized in Sangon (Sangon, Shanghai, China) and finally cloned into pNINC2EX.

### 4.3. Soybean Hairy Root Generation

Overexpressing and silencing plasmids were transferred into *Agrobacterium rhizogenes* K599 by the freeze-thaw method. Then, transgenic hairy roots were induced by the *A. rhizogenes*-mediated method described as Kereszt et al. [71]. Briefly, young seedlings with unfolded cotyledons are infected with *A. rhizogenes* at the cotyledonary node, and the infection sites are preserved in a humidified environment. After 5–7 days, hairy roots started to sprout from the site of infection. Hairy roots were covered with sterilized vermiculite wetted by Hoagland’s nutrient solution for ten days until hairy roots could sustain the plants. Then, the hairy roots were screened with a handheld lamp (Luyor, Shanghai, China) to visualize GFP expression. Hairy roots carrying strong GFP signals were reserved and used for further tests, and the rest of non-GFP hairy roots and the main roots were removed. After 30 days, transgenic soybean hairy roots were collected to confirm the effects of overexpressing and silencing by detecting the expression level of miR159 and target genes.

### 4.4. SCN Inoculation, Penetration and Development Evaluation

In order to inoculate soybean roots with SCN, J2s suspension was mixed with 0.2% water-agar equally to prepare the SCN inoculation suspension (final concentration of water-agar was 0.1%). 2000 J2s and 500 J2s were added to all the soybean plant roots and transgenic soybean hairy roots, respectively. In order to evaluate the penetration and development of SCN on W82, ten soybean roots of each treatment were stained with a boiling acid fuchsin solution [72]. Similarly, to evaluate penetration and development of SCN on transgenic hairy roots, ten roots per construct were used to evaluate penetration and development as described above. All nematode infection experiments were repeated at least two times.

### 4.5. Soybean gDNA, RNA Isolation and cDNA Synthesis and Quantitative Real-Time PCR (qRT-PCR) Analysis

Soybean cultivar Williams 82 seedlings roots were used for gDNA extraction with the NuClean Plant Genomic DNA Kit (CWbiotech, Beijing, China). SCN infected W82 soybean roots and W82 control roots were harvested for RNA extraction at 1 day post inoculation (dpi): 5 dpi, 10 dpi and 15 dpi. In total, there were three biological replicates, and each biological replicate consisted of three soybean roots used for RNA extraction and qRT-PCR. Total RNA was extracted with the Ultrapure RNA Kit (CWbiotech, Beijing, China). About 1 μg of total RNA was used to reverse transcription all cDNA of *GAMYB* genes with SYBR PrimeScript RT Master Mix kit (Takara, Dalian, China), and 2 μg of total RNA for miR159 cDNA synthesis with miRNA First Strand cDNA Synthesis kit (Sangon Biotech, Shanghai) was used.

All qRT-PCR experiments were performed on the CFX Connect Real-Time PCR Detection System (Bio-Rad, Herculesy, CA, USA). In order to examine *GAMYB* genes’ expression, SYBR Premix Ex Taq II kit (Takara, Dalian, China) was used to perform qRT-PCR, and soybean *ubiquitin 3* gene *GmUBI-3* (GenBank accession D28123.1) was used as an internal reference gene. For determining the expression of miR159, a universal reverse primer was used along with miR159 specific forward primers and in the MicroRNAs qPCR Kit (Sangon Biotech, Shanghai). Due to the poor melting curve and relatively lower amplification efficiency of miR159 subfamily members primers, the expression precursor of miR159 (pre-miR159) was also detected by using pre-miR159 specific forward primers and a universal reverse primer. Soybean U6 snRNA was used as the internal reference gene. In order to calculate the expression of miR159, pre-miR159 and *GAMYB* genes, the 2^−ΔΔCt^ method was applied to quantify qRT-PCR data [73]. All qRT-PCR reactions had three technical repeats, and results from one biological replicate are shown.

### 4.6. In Silico Analyses and Soybean miR159 Cleavage Sites Validation

The mature and precursor sequences of soybean miR159 family members were obtained from miRbase [74]. Phylogenetic analysis of *GAMYB* genes and precursor soybean gma-miR159 family members were implemented in MEGA-X [75] by using bootstrapped Maximum Likelihood (ML) estimation with 1000 bootstrap replications. The soybean mature miR159 sequences were placed into psRNATarget (http://plantgrn.noble.org/psRNAtarget/, accessed on 1 February 2020) to predict their target genes and cleavage sites with default parameters. Subsequently, 5’-RACE experiments were conducted to confirm the cleavage sites of miR159 with 5’-RACE RNA Kit (Sangon, Shanghai, China). Briefly, high quality and purity total RNA were extracted from SCN infected roots at 5 dpi and ligated with 5’-RACE RNA adaptor. Then, cDNA synthesis was performed using *GAMYB* specific outer primers, and nested PCR experiments were conducted. Finally, the PCR products were cloned into the T-vector and sequenced in Sangon (Sangon, Shanghai, China).

### 4.7. Chemical Treatment and Gibberellin Measurement

Gibberellic acid (GA_3_) was purchased from Sigma-Aldrich (Sigma-Aldrich, Shanghai, China). Before diluting in distilled water containing 0.02% (*v*/*v*) Tween 20, all chemicals were dissolved in a few drops of ethanol. An equal percentage of ethanol and Tween 20 was added into distilled water as a control treatment. Soybean roots were treated with four different concentrations of the following: GA_3_ (0.1, 1, 10 and 100 μM) for 12 h and hairy roots with 10 μM GA_3_ for 12 h. Sterile water was used to wash chemicals treated roots before nematode inoculation. For quantification of GA, SCN infected soybean roots and control root from three biological replicates, each consisting of a pool of three roots, were collected at 1, 5, 10 and 15 dpi; GA treated soybean roots and control roots were also collected from three biological replicates, each consisting of a pool of three roots after 12 h of GA treatment. The soybean roots were homogenized to powder with a grinder (Retsch, Shanghai, China). Next, an internal standard of GA was added into 50 mg powder of soybean roots and further extracted with 1 mL methanol/water/formic acid (15:4:1, *v*/*v*/*v*). Then, the extracted liquid was concentrated by using CentriVap Kansas, MO, USA) and redissolved with 80% methanol/water solution. Finally, the redissolved solution was filtered by a 0.22 μm filter membrane and transferred into a sample bottle for LC-MS/MS analysis (SCIEX, Redwood, CA, USA). In depth, Ultra Performance Liquid Chromatography ExionLC™ equipped with Waters ACQUITY UPLC HSS T3 C18 column (1.8 µm, 100 mm × 2.1 mm) was applied to separate the extracted GAs. Mobile phase: phase A, ultrapure water (containing 0.04% acetic acid); phase B, acetonitrile (containing 0.04% acetic acid). Gradient elution program: 0 min, phase A/phase B, 95:5 (*v/v*); 1.0 min, phase A/phase B, 95:5 (*v/v*), 8.0 min, phase A/phase B, 5:95 (*v/v*), 9.0 min, phase A/phase B, 5:95 (*v/v*), 9.1 min, phase A/phase B, 95:5 (*v/v*), 12.0 min, phase A/phase B, 95:5 (*v/v*); the flow rate was 0.35 mL/min; the column temperature was 40 °C; and the injection volume was 2 μL. Tandem Mass Spectrometry (MS/MS) QTRAP^®^ 6500+ was used to scan each ion pair basing on the optimized decluttering potential (DP) and collision energy (CE). The quantification of GA was conducted at Metware company (Metware, Wuhan, China).

### 4.8. Statistical Analysis

All statistical analyses were carried out using IBM SPSS STATISTIC v.22 (Armonk, NY, USA). The Kolmogorov–Smirnov test of normality (a = 0.05) was used to ensure that the data were normal, and all the analyses were parametric. In depth, independent *t*-test was used to detect the differences of relative expression levels of miR159 in comparison of W82 + SCN vs. W82CK, OE-miR159 vs. EV and STTM-miR159 vs. EV. Similarly, an independent *t*-test was also applied to detect the differences the differences of relative expression levels of *GmMYB33* genes in comparison of W82 + SCN vs. W82CK, OE-miR159 vs. EV and STTM-miR159 vs. EV. An independent *t*-test was used to detect the differences of GA content in comparison with W82 + SCN vs. W82CK at different time points, while the GA content in comparison with GA treated roots and control roots was analyzed by one-way analysis of variance (ANOVA) followed by LSD post hoc test. For analysis the SCN penetration and development data, one-way analysis of variance (ANOVA) followed by an LSD post hoc test was used to detect the differences in comparison of OE-miR159 vs. EV, GA treated roots vs. control roots and GA treated transgenic roots vs. EV.

## 5. Conclusions

In conclusion, the results from this study clearly indicate that soybean miR159 and target *GmMYB33* genes respond to the SCN infection. Transgenic soybean hairy roots harboring overexpressing miR159 plasmids could positively regulate soybean resistance to SCN by delaying the development of *SCN*In reverse, silencing miR159 reduced soybean resistance to *SCN*. Furthermore, exogenous application of GA enhanced the abundance of miR159 in soybean roots, suppressed the expression level *GmMYB33* genes and restricted the development of SCN. SCN infection results in endogenous GA content decreased significantly and lowered the expression of miR159. More importantly, exogenous application enhanced the soybean resistance to SCN in both wild type roots and miR159 silencing hairy roots. Overall, miR159-*GmMYB33* regulatory network participated in the GA modulated-resistance to SCN.

## Data Availability

All data and other materials can be obtained from authors.

## Supplementary Materials

The following are available online at https://www.mdpi.com/article/10.3390/ijms222313172/s1.

## Abbreviations

SCN	soybean cyst nematode
miRNA	microRNA
qRT-PCR	quantitative real-time PCR
dpi	day post inoculation
hpi	hour post inoculation
GA	Gibberellic acid
STTM	short tandem target mimics
