# Supplementary material for: Soybean miR159-GmMYB33 Regulatory Network Involved in Gibberellin-Modulated Resistance to Heterodera glycines
Source: Int J Mol Sci. 2021 Dec 6;22(23):13172. doi: 10.3390/ijms222313172 (PMC8658632; doi:10.3390/ijms222313172)
Supplement: Supplementary file 1 [file ijms-22-13172-s001.zip › ijms-1415615-supplementary.pdf]

Table S1. gma-miR159 family members described in miRbase

miRNA name	Location	Strand	Mature sequence-5p	Mature sequence-3p
gma-miR159a	chr9: 40266722-40266935	+	gagcuccuugaaguccaauug	uuuggauugaagggagcucua
gma-miR159b	chr7: 5424789-5424974	-	gaguucccugcacuccaaguc	auuggagugaagggagcucca
gma-miR159c	chr16: 2830034-2830218	-		auuggagugaagggagcuccg
gma-miR159d	chr9:40267067-40267127	+	agcugcuuagcuauggauccc	
gma-miR159e	chr7: 9561934-9562144	-	gagcuccuugaaguccaauu	uuuggauugaagggagcucua
gma-miR159f	chr16: 2819636-2819815	-	gaguucccugcacuccaaguc	auuggagugaagggagcucca

Table S2. Primers used in this research.

	Gene name		
	GmMYB33a RT primer	TGGAAATTGGGTGGTGAGTGCTTC	
	GmMYB33a inner primer	GCCTTTCAAGCTTTTCGAGTTGTGGACTATCGCTTCCAGC	
	GmMYB33a outer primer	CTGAAGCAGCAGATTGACCAAAGGGGGAATTTGGCTCCCC	
	GmMYB33b RT primer	GGTGCTTCATGCTTATCATCATGATCC	
	GmMYB33b inner primer	CGCTTCTAGCAAACCACTGCTTTGTGGAGAAATGGGATCTG	
	GmMYB33b outer primer	CCTAAGGGGGAATTTGGATCGCCTAGTTCATCCCATTCTGTC	
	GmMYB33f RT primer	GGTCTGAACTGGTAGCTGTTCGTTC	
	GmMYB33f inner primer	GTCTTTGCCTGATAAAGTAAAGCATCCAGCAGGCCAC	
	GmMYB33f outer primer	GCAGAGCTGTCAGCTCTGTCACCAGGGGTTGC	
	GmMYB33e RT primer	CTGGACGAGTAATATTCAACAGGGAC	
	GmMYB33e outer primer	CTCGTACACATTCAAAATAGAGCTGTCAGCTCTGTCACCAGGGG	
5′-RACE	GmMYB33e inner primer	CCGTTCAATGAATTTGCATTGGCTCTAAGAGCTGGGCACTC	
	GmMYB33d RT primer	GGATTGAAGTTGCTCCAAAGGGAG	
	GmMYB33d inner primer	GCATCAAGCAAGCCACTATTAAGTGGTGAAGAAGAACCCGAGTC	
	GmMYB33d outer primer	CAAAGCAGAACACTCAGCTCTGTCGCCAGGAG	
5′-RACE	GmMYB33c RT primer	GACTGCAATCTCTCATTGTTGCCAC	
	GmMYB33c inner primer	GTGGTGAAGAACCTGAGTCTAGTGCATTAGGTGGTTGAGG	
	GmMYB33c outer primer	AGCAGAACTCTCAGCTCTGTCACCAGGAGTTGC	
	Gene name	Forward	Reverse

Gene cloning	pre-miR159a	TAAGCAGGCGCGCCGAGGTTCATGGT CTTGGTGCTTTG	TAAGCACCTAGGAGGGTGTAGAGCTC CCTTCAATC
	pre-miR159b	TAAGCAGGCGCGCCGAATACCCTCTG GAGCTCCCTTC	TAAGCACCTAGGAAACCCAACTTGGA GTTCCCTG
	pre-miR159c	TAAGCAGGCGCGCCAAGGCCTAATTC GGAGCTCCC	TAAGCACCTAGGACCCAAGTTGGAGC TCTCTACAC
	pre-miR159e	TAAGCAGGCGCGCCACAAAAGGGGA GAAGGGTGTAGAG	TAAGCACCTAGGCAAAGGGGGTTATG GAGTGGAGC
	pre-miR159f	TAAGCAGGCGCGCCACCCTCTGGAGC TCCCTT	TAAGCACCTAGGACCCAAGTTGGAGT TCCCTGC
	Gene name	Forward	Reverse
qRT-PCR	U6	GGAACGATACAGAGAAGATTAGCA	TTTGGACCATTTCTCGAT
	pre-miR159a	GAAGTGGAGCTCCTTGAAGTCC	Universal
	pre-miR159b	GGAGTTCCCTGCACTCCAAGT	Universal
	pre-miR159c	GGAGCTCTCTACACTCCAAGTCT	Universal
	pre-miR159e	CAAAGGGGGTTATGGAGTGGAGC	Universal
	pre-miR159f	ACCCAAGTTGGAGTTCCCTGC	Universal
	miR159a/e-5p	CAGGAGCTCCTTGAAGTC	Universal
	miR159a/e-3p	GCGCAGTTTGGATTGAAG	Universal
	miR159b/f/-5p	GAGTTCCCTGCACTCCA	Universal
	miR159b/f/-3p/c	GCGCAGATTGGAGTGAAG	Universal
	GmMYB33b	CTCGGGAGTCAGAAATGCTATAC	CAACCCAGGATGGTCAGAAA
	GmMYB33f	TGCTCGAGTCAGTTGATGATTT	CATCCAGCAGGCCACTATTT
	GmMYB33e	GCAGAAGAGGAGCGGCTGATTG	CTGTCCGACCAGGCAAATGAGC
	GmMYB33d	CTGCGATGGGCCAATCACCTAAG	TGGCGTGGAGTTCAGCAATCATC
	GmMYB33c	GGAAACAAATGGGCACGCATGG	CTCGGGAGGATAAAGTGGCAAGC
	GmMYB33a	AGACAGTTCCAGGAAGAAA	ACTTCCATACTCGGTGCTATTC
	GmUBI-3	GTGTAATGTTGGATGTGTTCCC	ACACAATTGAGTTCAACACAAACCG
STTM-miR159			
miR159a/e-3p	GGCGCGCCGTAGAGCTCCCTCTATCAATCCAAAGTTGTTGTTGTTATGGTCTAATTTAAATATGGTCT AAAGAAGAAGAATTAGAGCTCCCTCTATCAATCCAAACCTAGG		
miR159b/f-3p/c	GGCGCGCCTGGAGCTCCCTCTATCACTCCAATGTTGTTGTTGTTATGGTCTAATTTAAATATGGTCTA AAGAAGAAGAATTGGAGCTCCCTCTATCACTCCAATCCTAGG		

Note: red characters indicate restriction enzyme sites.

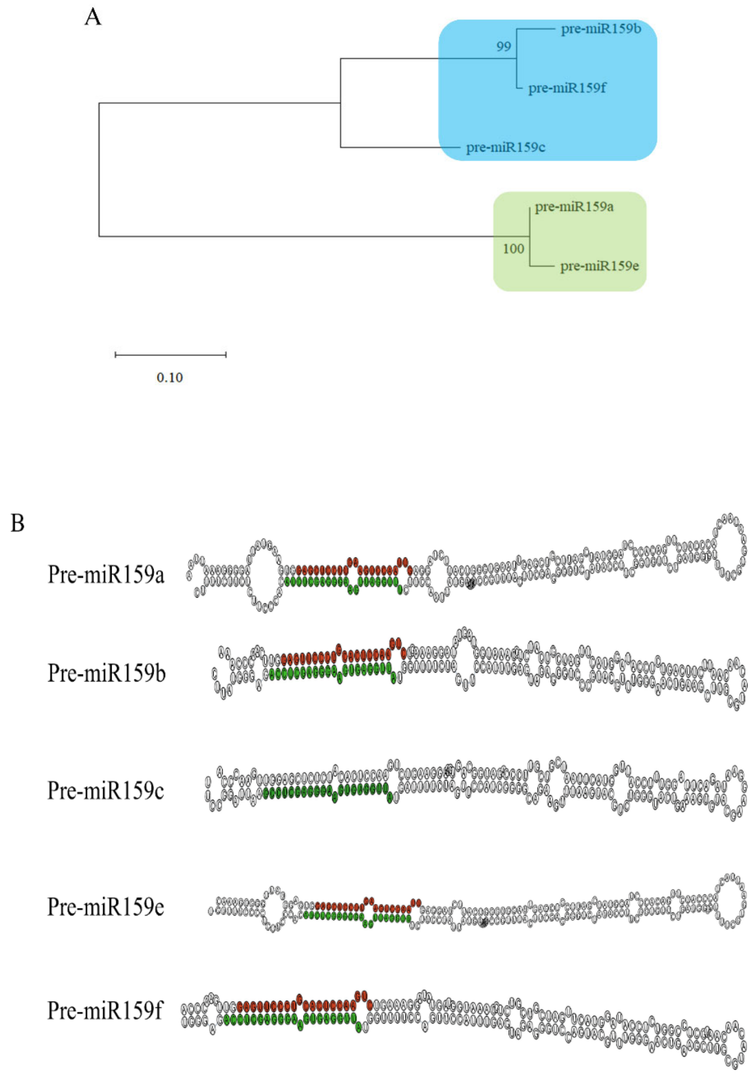

Figure S1. The soybean MIR159 gene family. A. Phylogenetic analysis of gma-miR159 family. Multiple sequences alignments were generated with Clustal W, using default parameters in MEGAX. Phylogenetic analysis was performed in MEGAX using bootstrapped Maximum Likelihood (ML) estimation with 1000 bootstrap replications. B. The stem-loop structures of precursors of gma-miR159 family members. miR159-5p and miR159-3p are colored with red and green. The sequences of precursors of gma-miR159 were downloaded from miRbase and folded by using RNAfold wrapper in TBtools.

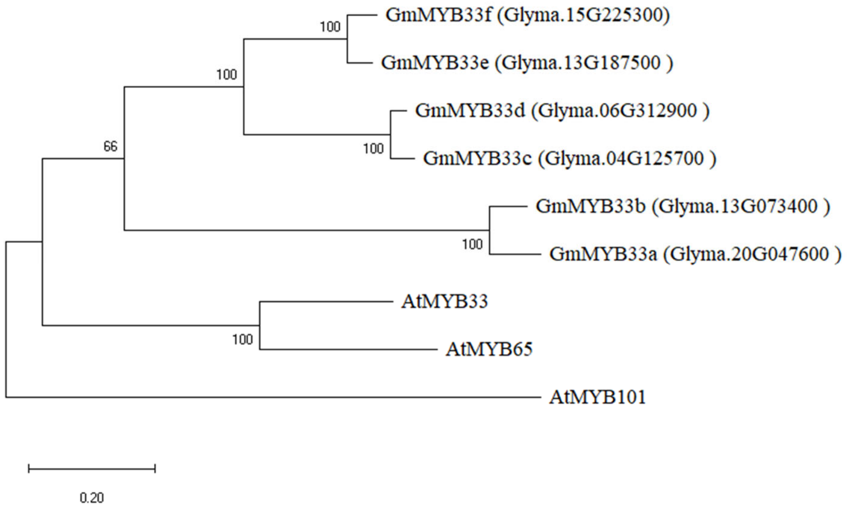

Figure S2. Phylogenetic analysis of soybean miR159 target *GAMYB* genes. The protein sequences of *GAMYB* genes were downloaded from Soybase and used for multiple sequences alignments with MUSCLE wrapper. Phylogenetic analysis was performed in MEGAX using bootstrapped Maximum Likelihood (ML) estimation with 1000 bootstrap replications.

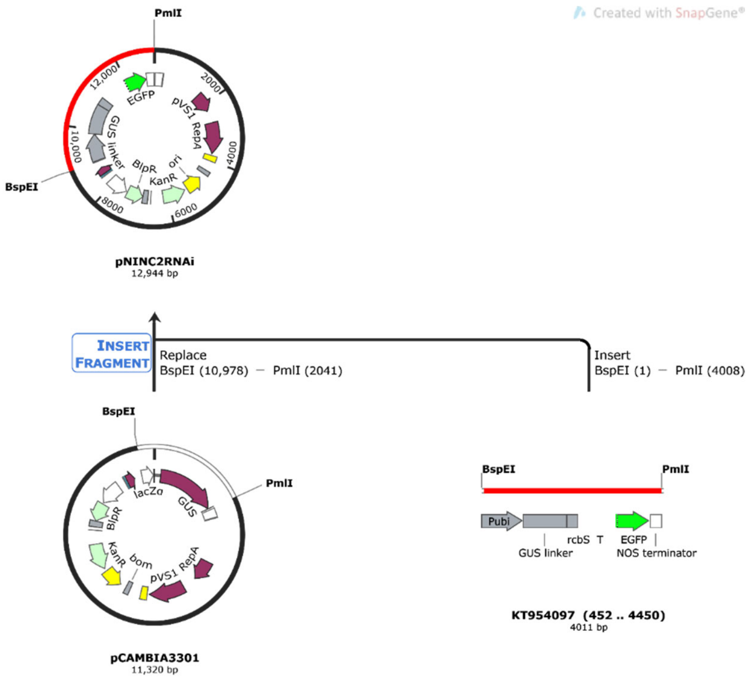

Figure S3. Schematic diagram of construction of pNINC2RNAi plasmid. The expression part of pG2RNAi2 (GenBank: KT954097, from 452th to 4450th nucleotide, 5′ end flanked with BspEⅠ and 3′ end flanked with PmlⅠ restriction enzyme sites) that contains *Gmubi* promotor, GUS(β-glucuronidase) linker, rcbS terminator and an EGFP (enhanced Green Fluorescent Protein) expression cassette, then, the synthesis part was inserted into pCMBIA3301 by restriction digest clone. The new binary plasmid was named pNINC2RNAi.

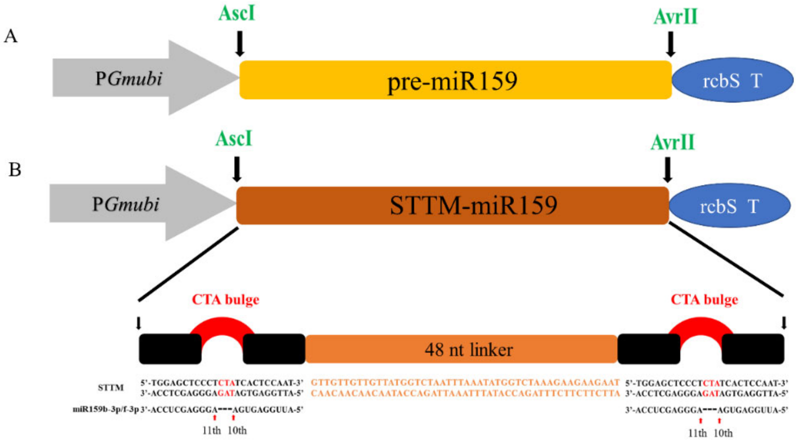

Figure S4. Schematic diagram of gene cassette used for the overexpression and silencing of miR159 in soybean hairy roots. A, schematic diagram of overexpressing pre-miR159, B, schematic diagram of silencing miR159, STTM159 consists of two short identical sequences that mimic miR159 target sites with a three additional nucleotides CTA bulge corresponding to the positions 10 to 11 of the miRNA159. Two short identical sequences were linked with 48 nt linker, mature miR159b-3p/f-3p sequence was used as example. PGmubi, Gmubi promotor, rcbS T, rcbS terminator, AscⅠ, AvRⅡ indicate the AscⅠ and AvRⅡ restriction enzyme sites, respectively.

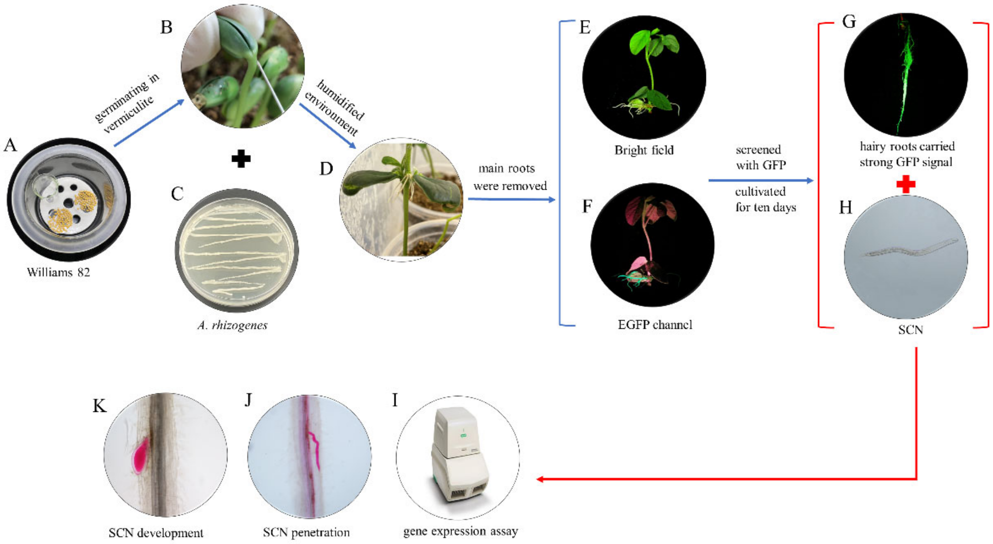

Figure S6. Generation of transgenic soybean hairy roots and SCN development on gene overexpressing and silencing hairy roots. A, soybean cultivar Williams 82 seeds, B, Williams 82 seedlings, C, *Agrobacterium rhizogenes* contained overexpressing and silencing plasmids, D, hairy roots started to sprout from the site of infection of *Agrobacterium rhizogenes*, E, bright field of transgenic soybean hairy roots, F, EGFP channel of transgenic soybean hairy roots, G, hairy roots carried with strong GFP signal, H, freshly hatched SCN juveniles, I, gene expression level assay with CFX Connect Real-Time PCR Detection System (Bio-Rad, CA, USA), J, K, SCN juveniles penetrate and develop in hairy roots, respectively.

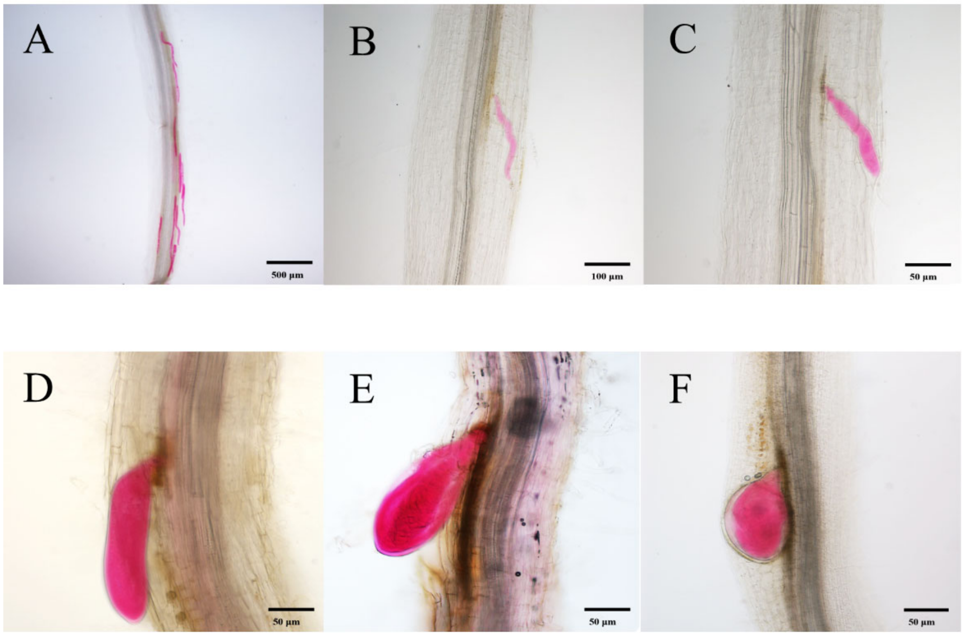

Figure S6. Different stage juveniles of soybean cyst nematode in soybean root. A: Second-stage juvenile, B: Swollen second-stage juvenile, C: Third-stage juvenile, D: Fourth-stage juvenile, E: Young female, F: Adult Female. Due to the progression from late J2 to the third-stage juvenile (J3), fourth-stage juvenile (J4) and to adult females is very rapid in the soybean roots, which makes it difficult to distinguish the late J2 stage from J3/J4 and the J4 from early female stage by using a stereo microscope. To evaluate the nematode development in a very consistent manner, we applied a three-grade scoring system consisting of the second-stage juvenile (veriform-J2, A), swollen juveniles (swollen J2/J3/J4, B, C, D), young females (E) and mature females (F).
